# Pancreatic Acinar Cell Carcinoma in a Patient with Familial Adenomatous Polyposis Who Achieved Long-Term Survival after Repeated Resection of Liver Metastases: A Case Report

**DOI:** 10.70352/scrj.cr.26-0605

**Published:** 2026-10-28

**Authors:** Ayano Takahashi, Toshiro Ogura, Naganori Yamada, Koichiro Kumano, Ibuki Fujinuma, Hiroaki Kanda, Amane Takahashi

**Affiliations:** 1 Department of Gastroenterological Surgery, Saitama Cancer Center, Ina-machi, Saitama, Japan; 2 Department of Pathology, Saitama Cancer Center, Ina-machi, Saitama, Japan

**Keywords:** pancreatic acinar cell carcinoma, liver metastasis, hepatectomy, familial adenomatous polyposis

## Abstract

**INTRODUCTION:**

Pancreatic acinar cell carcinoma (PACC) is a rare exocrine pancreatic neoplasm that differs from pancreatic ductal adenocarcinoma (PDAC) in its histogenetic origin and oncological behavior. Although recurrence is common after curative-intent resection, the optimal management of recurrent PACC remains undefined. Furthermore, although a certain number of pancreatic tumors have been reported in association with familial adenomatous polyposis (FAP), the relationship with PACC in particular remains poorly understood. The present case may help address this issue.

**CASE PRESENTATION:**

At age 22, the patient underwent distal pancreatectomy for a pancreatic tumor, which was diagnosed as PACC. A local recurrence occurred in the fourth postoperative year and achieved complete response with chemotherapy. From the tenth postoperative year onward, the patient developed repeated liver metastases, for which a total of 5 hepatectomies were performed; the patient has remained recurrence-free for 72 months since the last surgery. During the clinical course, a pathogenic adenomatous polyposis coli (*APC*) variant was identified, leading to a definitive diagnosis of FAP.

**CONCLUSIONS:**

We experienced a case in which an aggressive surgical approach—repeated hepatectomy for multiple recurrent liver metastases from PACC—achieved long-term recurrence-free survival. This supports active surgical resection as a viable strategy for PACC liver metastases. Furthermore, this rare case of PACC arising in a patient with FAP also raises the hypothesis that the APC/β-catenin pathway may be biologically involved in PACC tumorigenesis; however, a single case cannot establish an epidemiological association between FAP and PACC incidence, and further investigation is warranted.

## Abbreviations

AJCC
: American Joint Committee on Cancer
APC
: adenomatous polyposis coli
CA19-9
: carbohydrate antigen 19-9
CEA
: carcinoembryonic antigen
CR
: complete response
CS
: colonoscopy
DUPAN-2
: Duke pancreatic monoclonal antigen type 2
EGD
: esophagogastroduodenoscopy
FAP
: familial adenomatous polyposis
Gd-EOB-MRI
: gadolinium-ethoxybenzyl-diethylenetriamine pentaacetic acid–enhanced MRI
GS
: gemcitabine plus S-1
LST
: laterally spreading tumor
NSE
: neuron-specific enolase
PACC
: pancreatic acinar cell carcinoma
PAS
: periodic acid–Schiff
PDAC
: pancreatic ductal adenocarcinoma
SSTR2
: somatostatin receptor type 2
UICC
: Union for International Cancer Control

## INTRODUCTION

PACC is a rare pancreatic exocrine neoplasm, accounting for an estimated 0.4%–1.0% of all pancreatic tumors.^1)^ It differs from PDAC in its histogenetic origin and oncological behavior. The prognosis after curative resection is more favorable than that of PDAC, and surgical resection is considered the first-line treatment; however, no consensus has been established regarding treatment for recurrent disease. Reports of long-term survival achieved through repeated hepatectomy for multiple recurrent liver metastases remain extremely limited.

FAP is a hereditary tumor syndrome caused by *APC* gene mutations and is associated with a wide range of malignancies. Regarding pancreatic tumors, a previous cohort study reported a cumulative incidence of PDAC of 0.7% by age 70 and a relative risk of 2.2 in patients with FAP.^2)^ In contrast, epidemiological data on PACC are lacking owing to its rarity; however, PACC and FAP share *APC* gene abnormalities in their pathogenesis, and an association between the 2 cannot be excluded.

We herein report a case in which the patient developed recurrent liver metastases after pancreatic resection for PACC and achieved long-term survival through a total of 5 hepatectomies. Reports of PACC arising in a patient with FAP, let alone cases in which repeated surgery was performed for recurrent liver metastases from PACC, are exceedingly rare. This case highlights the potential role of repeated resection in selected patients with recurrent PACC and raises the possibility of an association between PACC and FAP.

## CASE PRESENTATION

At age 22 (May of year X), he presented to a previous hospital with fever and abdominal pain. His medical history was notable for resection of a juvenile nasopharyngeal angiofibroma at age 17. His father and paternal grandfather had reportedly been diagnosed with FAP and had died of colorectal cancer. Further examination revealed a 10-cm multilocular cystic tumor in the pancreatic body and tail. In June of the same year, he underwent distal pancreatectomy, splenectomy, and combined resection of the transverse colon as a combined diagnostic and therapeutic procedure. Histopathological examination of the resected specimen (**Fig. 1A**, **1B**) showed tumor cells with round to slightly polygonal nuclei and scant cytoplasm proliferating in sheets, focally forming acinar structures, with numerous mitotic figures. PAS staining showed granular PAS-positive material in part of the tumor. Immunohistochemistry showed positivity for Bcl-10 (**Fig. 1C**); membranous and nuclear positivity for β-catenin; positivity for CAM5.2; focal positivity for AE1/AE3, CD10, and NSE; and negativity for synaptophysin, CD56, chromogranin A, SSTR2, S-100, insulin, and p53. The final diagnosis was PACC (medullary type, INFα, Ly0, V0, ne0, mpd (−), PCM (−), DPM (−), R0, pT2N0M0, pStage II according to the 5th edition of the Japanese Classification of Pancreatic Cancer; pT2N0M0, pStage IB according to the 6th edition of the AJCC/UICC TNM classification, contemporaneous with the time of surgery).

**Fig. 1:**
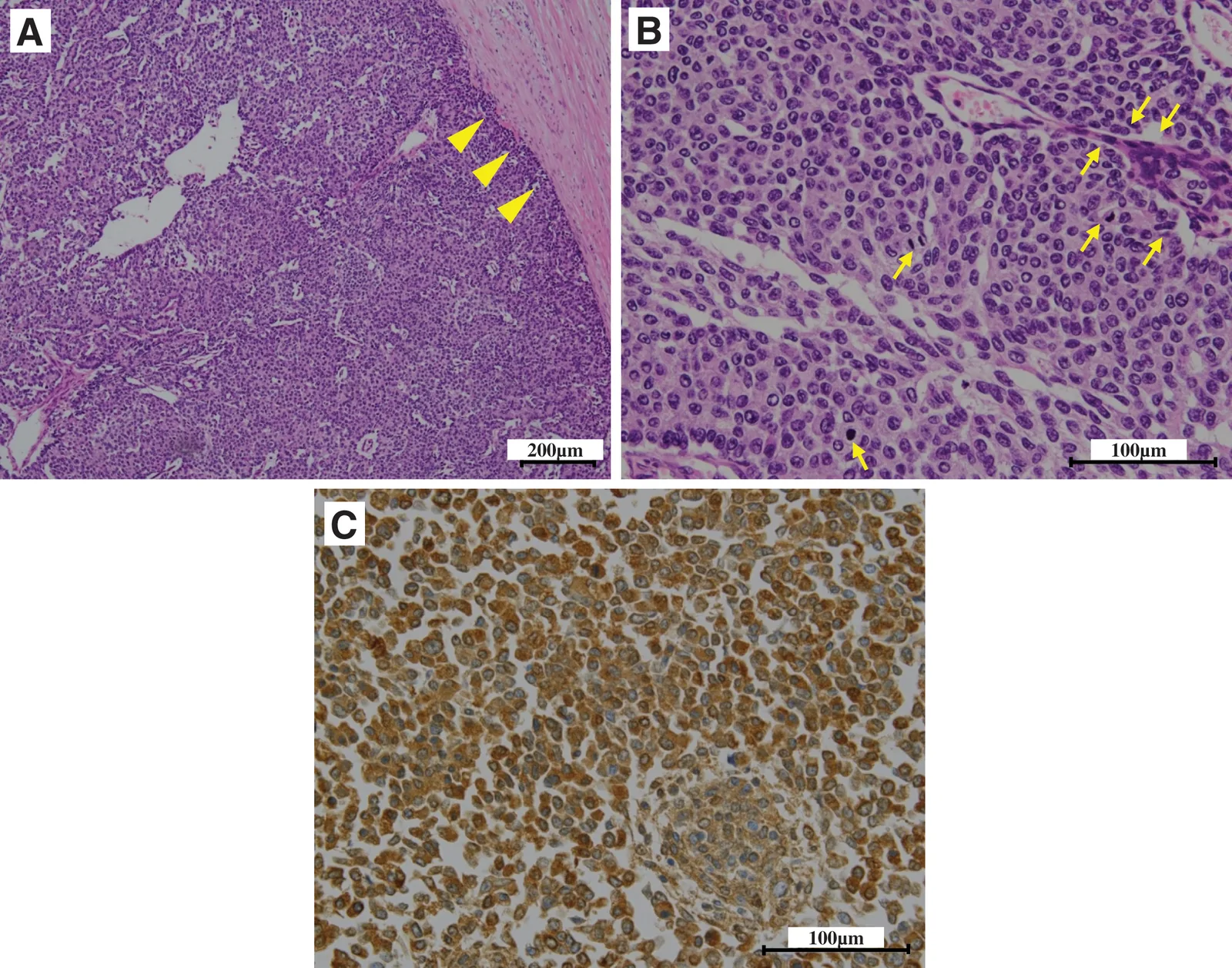
Histopathology findings of the initial resection specimen. (**A**) HE staining shows a thick capsule (arrowheads); tumor cells with round-polygonal nuclei and scant cytoplasm proliferate in sheets, focally acinar. (**B**) High power shows marked nuclear atypia with numerous mitoses (arrows), 27/10 HPF. (**C**) Immunohistochemically, the tumor cells showed cytoplasmic positivity for Bcl-10. HE, hematoxylin and eosin; HPF, high-power field

EGD and CS performed before the initial surgery revealed multiple polyps, and based on this finding together with the family history, the patient was clinically diagnosed with FAP. He did not undergo genetic testing at that time, and surveillance CS was performed annually.

In year X + 4, a suspected local recurrence was identified (**Fig. 2A**). This local recurrence was diagnosed clinically at the referring hospital based on imaging findings alone, as biopsy was not performed; detailed imaging and laboratory data from that period are not available. It was judged unresectable, and GS therapy followed by S-1 monotherapy was administered; the patient was referred to our hospital in September of year X + 5.

**Fig. 2:**
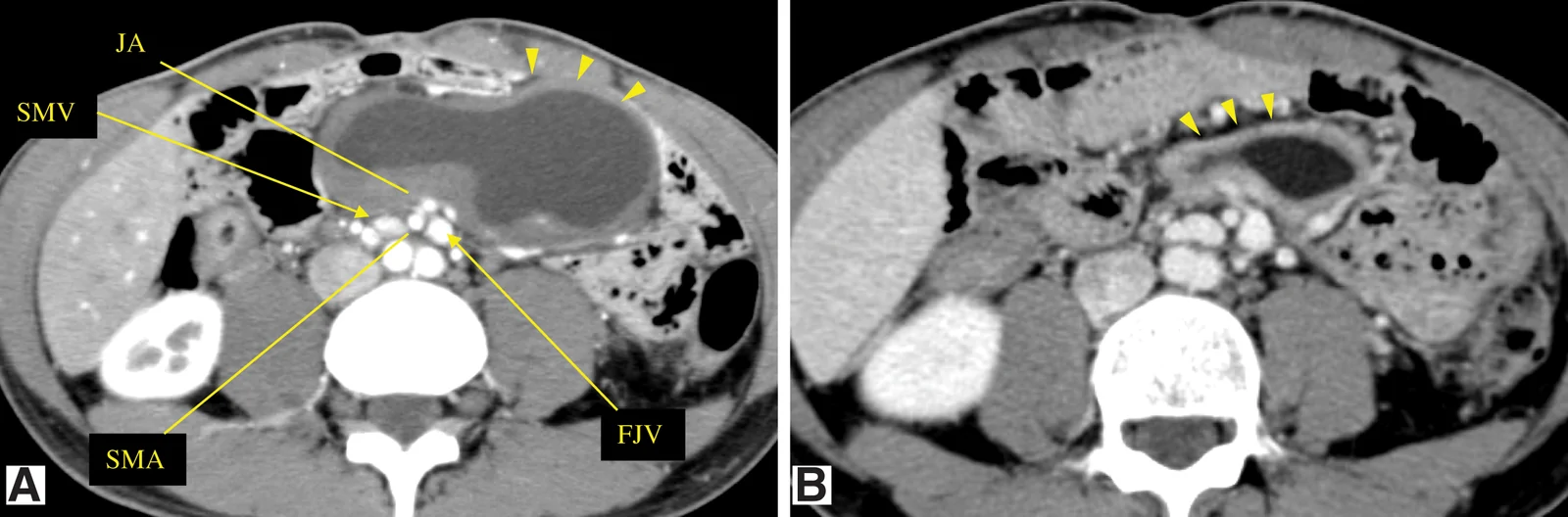
CT images of the locally recurrent lesion before and after chemotherapy. (**A**) A thick-walled cystic lesion (arrowheads) at the jejunal mesenteric root was thought to be a local recurrence. This lesion was abutting the SMA, JA, SMV, and FJV, deemed unresectable. (**B**) After chemotherapy, the lesion had shrunk, consistent with clinical CR. CR, complete response; FJV, first jejunal vein; JA, jejunal artery; SMA, superior mesenteric artery; SMV, superior mesenteric vein

Tumor shrinkage was observed, and S-1 therapy was continued. The tumor shrinkage was maintained thereafter (**Fig. 2B**), and in September of year X + 6, a clinical CR was confirmed based on the radiological disappearance of the enhancing lesion on contrast-enhanced imaging, which was sustained on serial follow-up studies. Chemotherapy was discontinued at that point.

In June of year X + 10 (10 years 0 months after the initial surgery), contrast-enhanced CT (**Fig. 3**) revealed recurrent liver metastases (segments 6 and 7). The site previously judged to be a clinical CR after chemotherapy showed resolution of the cystic component but a persistent thick wall, and it was resected simultaneously. Partial hepatectomy (2 sites in segments 6 and 7) and partial jejunal resection at the site of the local recurrence were performed. Histopathological examination confirmed that the hepatic lesions (segment 6, 13 mm; segment 7, 15 mm) were metastases from PACC. The jejunal mesenteric lesion showed no definite malignant findings and was considered post-chemotherapy scar tissue.

**Fig. 3:**
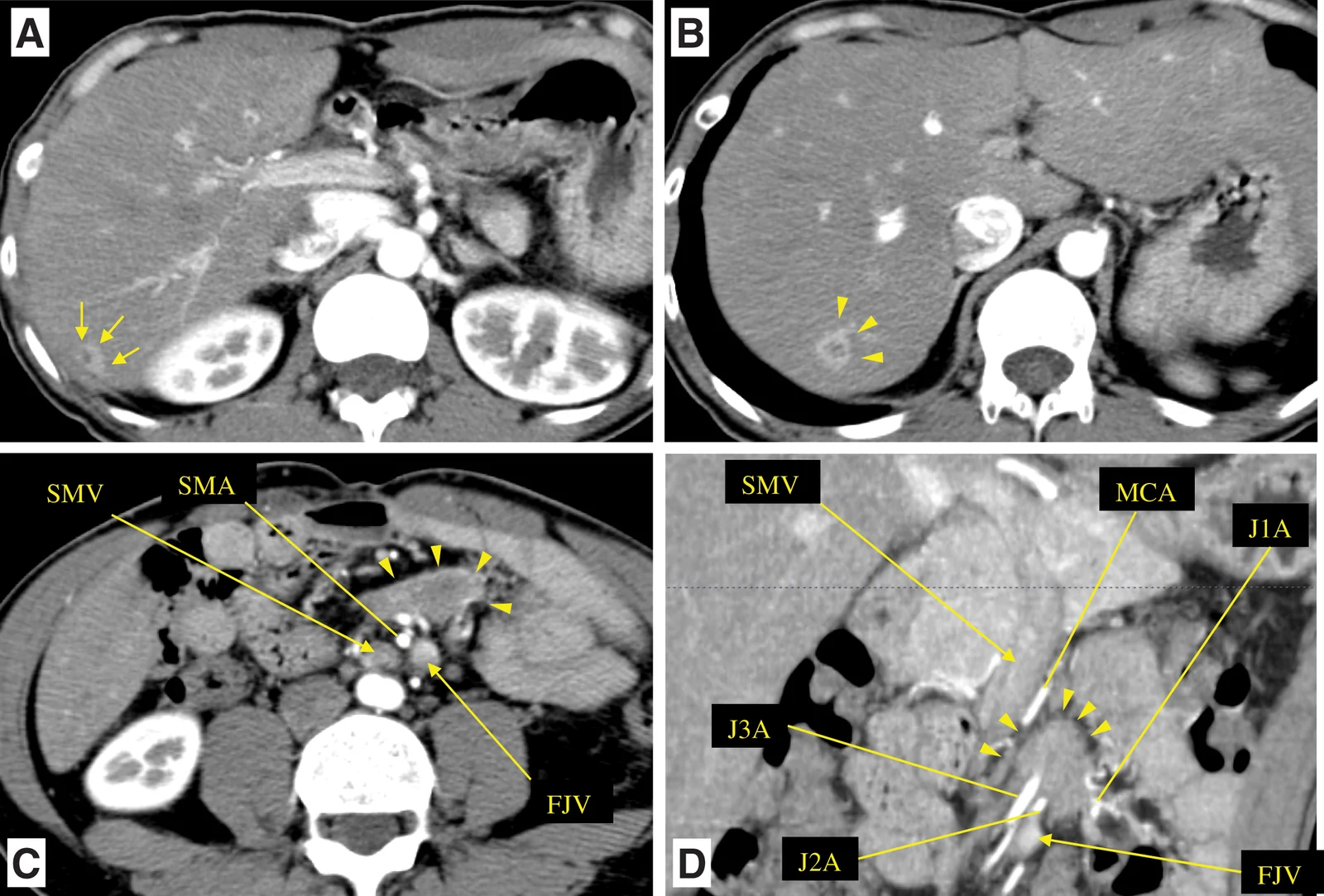
CT imaging findings at the time of the second recurrence. (**A**, **B**) Arterial-phase ring-enhancing masses in segments 6 (arrows) and 7 (arrowheads), suggesting hepatic recurrences. (**C**, **D**) On axial images, the jejunal mesenteric lesion showed further loss of internal fluid after chemotherapy, leaving only a thick capsule (arrowheads). On coronal images, the lesion remained close to critical vessels; however, it had decreased in size, and resection was performed. FJV, first jejunal vein; J1A, first jejunal artery; J2A, second jejunal artery; J3A, third jejunal artery; MCA, middle colic artery; SMA, superior mesenteric artery; SMV, superior mesenteric vein

In November of year X + 11 (11 years 5 months postoperatively), CT (**Fig. 4A**) revealed recurrence in liver segment 6, and partial hepatectomy of segment 6 was performed; this was the second hepatectomy. Histopathological examination again confirmed liver metastasis from PACC, as in the initial recurrence.

**Fig. 4:**
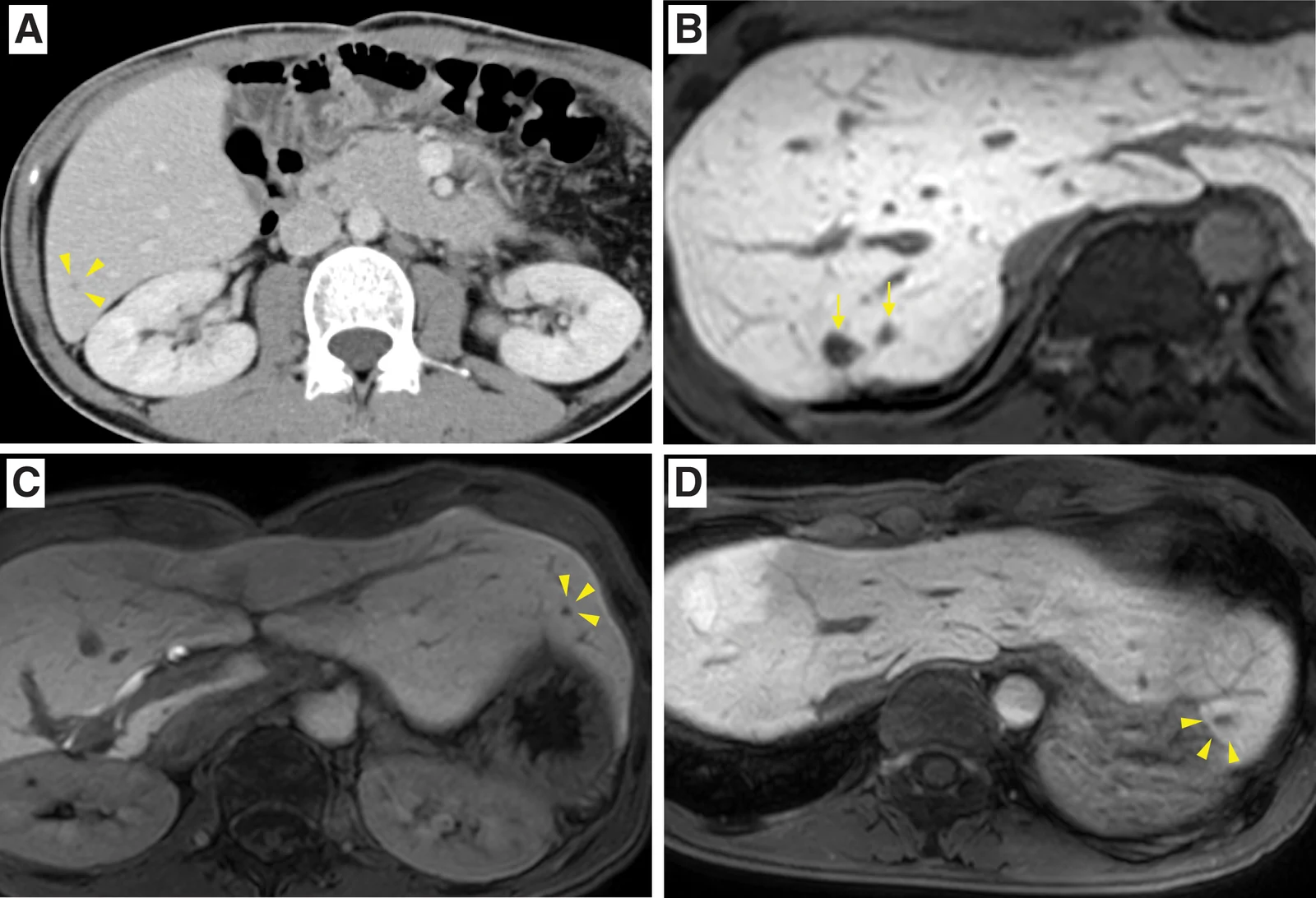
Imaging findings at each recurrence from the third episode onward. (**A**) 3-mm low-density area (arrowheads) in segment 6, suggestive of the second hepatic recurrence. (**B**) On the hepatobiliary phase of Gd-EOB-MRI, masses showing low signal intensity (arrows) are observed in the posterior segment of the liver, consistent with the third hepatic recurrence. The recurrence involved multiple hepatic metastases localized within the posterior segment, including the 2 lesions shown in this representative slice. (**C**) On the hepatobiliary phase of EOB-MRI, a mass showing low signal intensity appeared in liver segment S3 (arrowheads), consistent with the fourth hepatic recurrence. (**D**) On the hepatobiliary phase of EOB-MRI, a mass showing low signal intensity appeared in liver segment S2 (arrowheads), consistent with the fifth hepatic recurrence. Gd-EOB-MRI, gadolinium-ethoxybenzyl-diethylenetriamine pentaacetic acid–enhanced MRI

In April of year X + 12 (11 years 10 months postoperatively), Gd-EOB-MRI (**Fig. 4B**) revealed multiple liver metastases, and posterior sectionectomy of the liver was performed. Histopathological examination confirmed liver metastases from PACC, with a total of 14 metastatic foci identified, the largest measuring 14 mm.

In April of year X + 13 (12 years 10 months postoperatively), Gd-EOB-MRI (**Fig. 4C**) revealed a metastasis in liver segment 3, and partial hepatectomy of segment 3 was performed. Histopathological examination confirmed liver metastasis from PACC.

In May of year X + 17 (16 years 11 months postoperatively), Gd-EOB-MRI (**Fig. 4D**) revealed a metastasis in liver segment 2, and partial hepatectomy of segment 2 was performed. Histopathological examination (**Fig. 5**) again confirmed liver metastasis from PACC, as in the previous specimens. Pathological R0 resection was achieved in all 5 hepatectomies (**Fig. 6A**–**6F**). In the third hepatectomy (posterior sectionectomy, **Fig. 6D**), one of the tumors was exposed at the resection margin (0-mm margin resection), but there was no incision into the tumor itself, and the resection was therefore judged as R0.

**Fig. 5:**
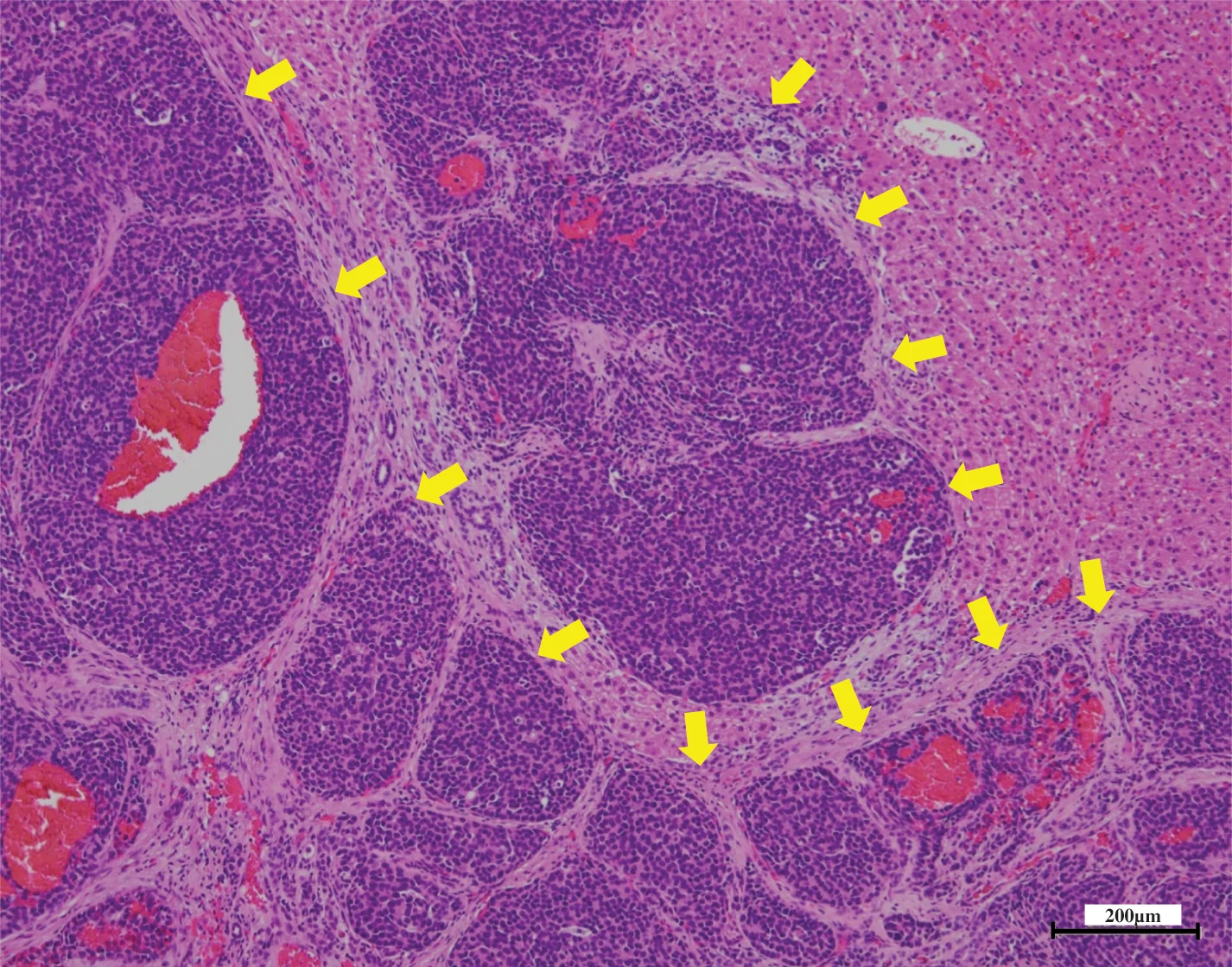
Histopathological findings of the resected specimen at the fifth hepatectomy. HE staining showing atypical tumor cells proliferating with acinar-like structures (thick arrows), consistent with previous specimens. HE, hematoxylin and eosin

**Fig. 6:**
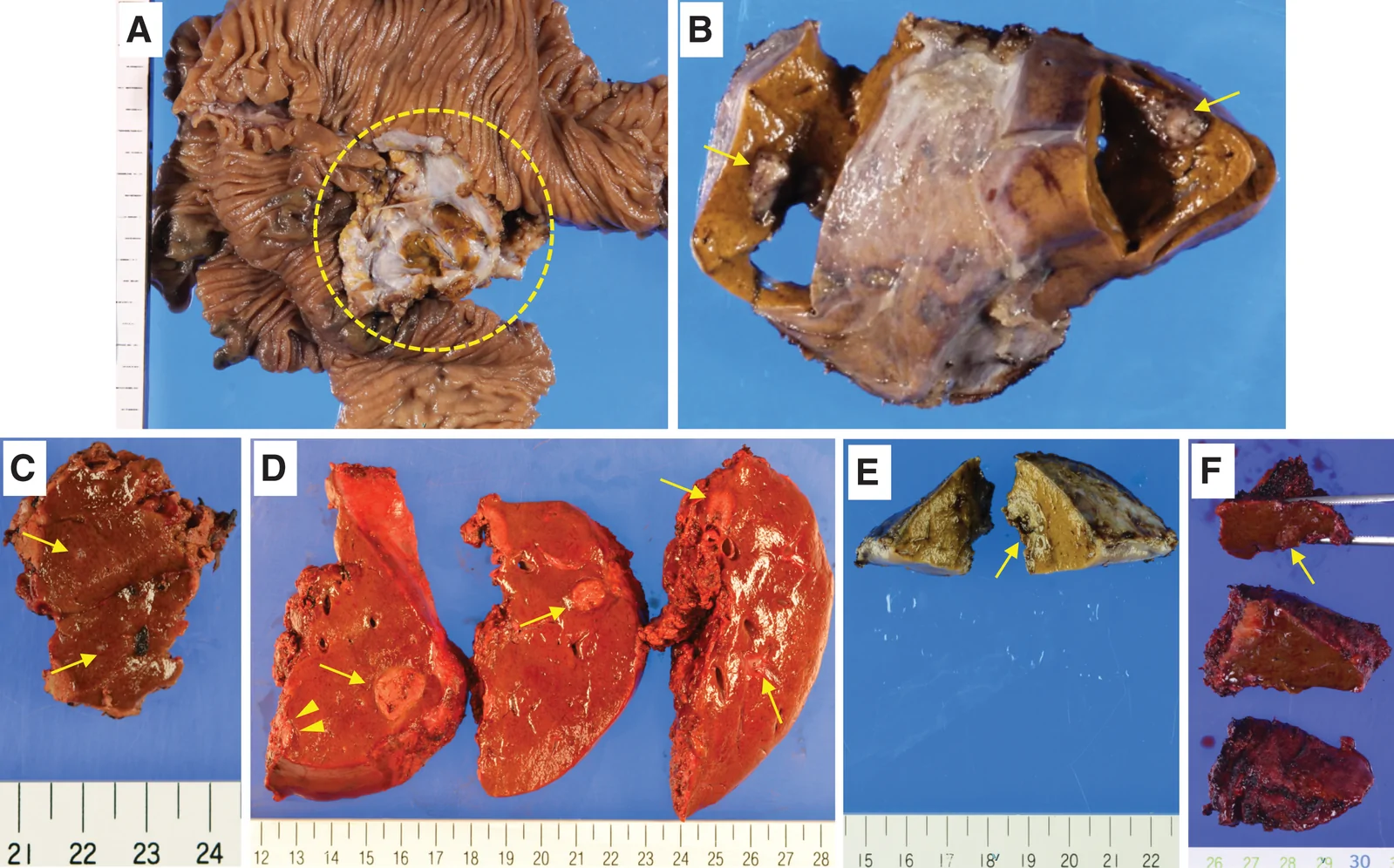
Photographs of the resected specimens from the 5 hepatectomies performed for recurrent liver metastases. (**A**, **B**) The first surgery for recurrence: (**A**) Specimen of the resected local recurrence, together with the concomitant partial jejunectomy. (**B**) Liver resection specimen from the first hepatectomy (arrow, tumor). (**C**) Second hepatectomy: arrows, tumor. (**D**) Third hepatectomy: arrows, tumor; arrowheads, tumor exposed at the resection margin (0-mm margin). (**E**) Fourth hepatectomy: arrow, tumor. (**F**) Fifth hepatectomy: arrow, tumor. Pathological R0 resection was achieved in all 5 hepatectomies; in the third hepatectomy, one of the tumors was exposed at the resection margin (0-mm margin) at the site indicated by the arrowhead in (**D**), although there was no incision into the tumor itself.

The patient has since been followed with regular CT, MRI, and tumor markers (CEA, CA19-9, and DUPAN-2), all of which remained within normal limits without findings suggestive of recurrence, and has remained recurrence-free for 72 months since the last surgery.

During this clinical course, genetic testing performed in December of year X + 17 revealed a germline pathogenic *APC* variant (c.3214_3220dup), confirming the diagnosis of FAP. The patient has since undergone surveillance CS approximately twice yearly (12 examinations in total), and repeated endoscopic resection has been performed for multiple polyps and LSTs identified during follow-up. The patient has been able to avoid prophylactic total colectomy to date. A chronological summary of this clinical course is shown in **Fig. 7**.

**Fig. 7:**
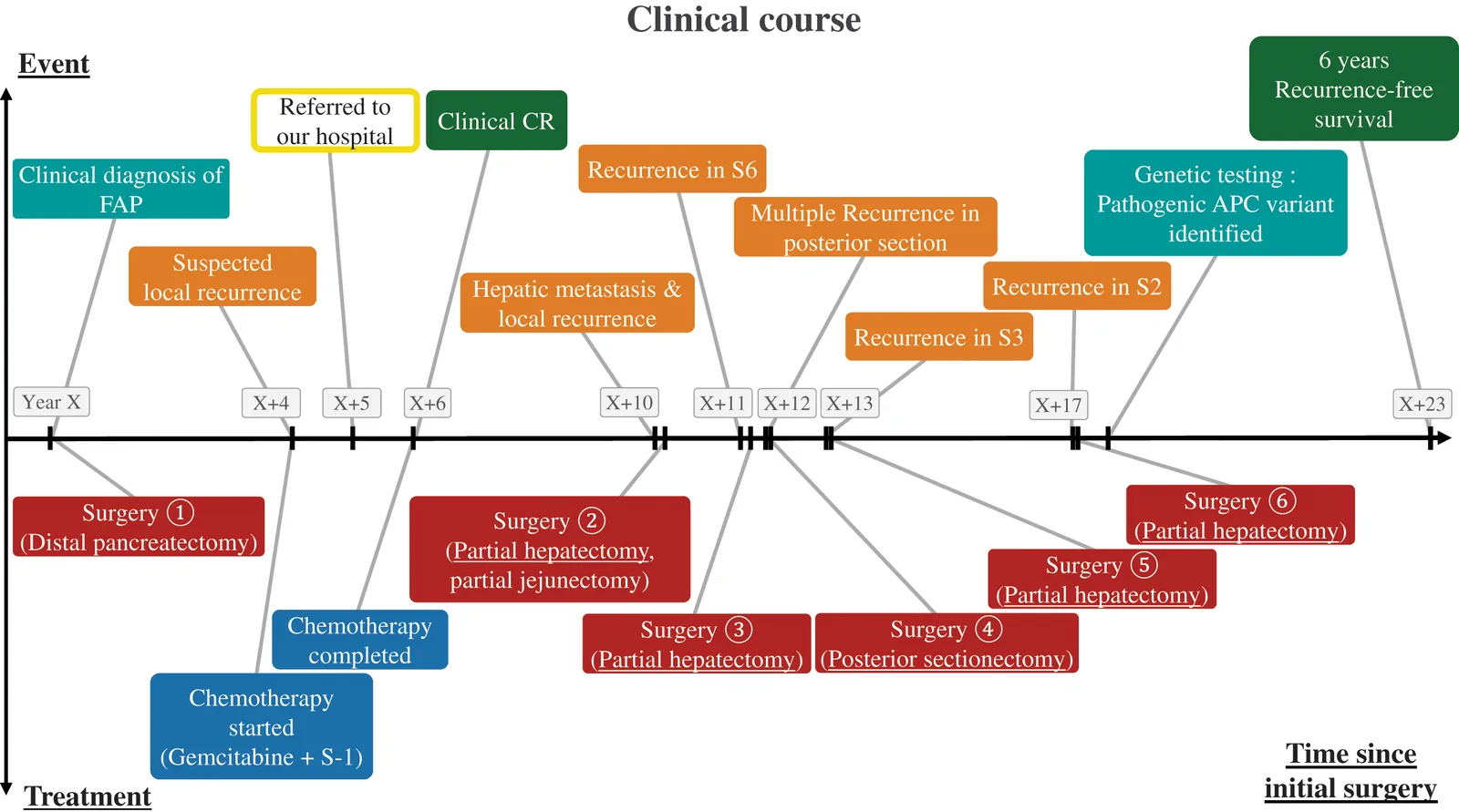
Clinical course of the patient. Chronological summary of surgical treatments. Surgery 1 was the initial distal pancreatectomy; surgeries 2–6 were hepatectomies for successive recurrent hepatic metastases, at the intervals shown. The patient has remained recurrence-free for 72 months since Surgery 6. APC, adenomatous polyposis coli; CR, complete response; FAP, familial adenomatous polyposis

## DISCUSSION

This case involved a patient with underlying FAP who developed PACC and, following the appearance of multiple liver metastases from the tenth postoperative year onward, achieved long-term survival through repeated hepatectomy. Reports of repeat hepatectomy for recurrent liver metastases from PACC and reports of PACC arising in patients with FAP are both scarce. To our knowledge, this is the first report describing the treatment course of liver metastases from PACC in a patient with FAP.

PACC is an extremely rare tumor among pancreatic exocrine neoplasms, accounting for a reported 0.4%–1.0% of all pancreatic tumors^1)^ and 1%–2% of pancreatic exocrine tumors.^3)^ PACC differs substantially from PDAC in both clinical presentation and biological behavior. Wisnoski et al.^4)^ analyzed 672 patients with PACC and reported an overall 5-year survival rate of 42.8% (median survival, 47 months), 71.6% (median survival, 123 months) among patients undergoing curative resection, and 22% (median survival, 25 months) among those who did not undergo resection. Kitagami et al.^1)^ similarly examined 115 patients with PACC and reported a 5-year survival rate of 43.9% (median survival, 41 months) among resected patients (n = 87) and 0% (median survival, 4 months) among unresected patients; among the resected patients, 11 developed recurrences, most commonly in the liver (8 cases). Although the reported outcomes vary, the prognosis of PACC is generally more favorable than that of PDAC, with a marked survival benefit observed after curative resection, and surgical resection is considered the first-line treatment when local control is achievable.^3)^ The recurrence rate after resection is high and frequently involves the liver. No consensus exists regarding treatment for recurrence; while reports describe disease control achieved with chemotherapy or a single hepatectomy for recurrent liver metastases, reports of repeated hepatectomy for multiple recurrences are limited. Only 3 prior reports^5–7)^ have described repeated resection for recurrent liver metastases from PACC, as in the present case (**Table 1**; ages shown are at the time of initial diagnosis). Compared with these reports, the present case is notable for its higher number of liver recurrences (5 episodes), each of which was managed by hepatectomy, achieving a recurrence-free survival of 72 months after the last treatment. These findings suggest that repeated surgical resection is a reasonable treatment option for recurrent liver metastases from PACC when local control can be achieved. Nonetheless, given that this patient experienced recurrence repeatedly over an approximately 17-year clinical course, the current 72-month recurrence-free interval cannot be regarded as a definitive cure, and continued long-term surveillance remains essential.

**Table 1:** Repeated hepatectomy for liver metastasis from PACC

Author, year	Age, sex	Initial surgery	Time from initial surgery to metastasis (months)	Site of recurrences	No. of liver metastases	Treatment strategy	Total no. of hepatectomy	RFS (months)
Hashimoto, 2007^7)^	61, F	DP + left hepatectomy + partial hepatectomy	1st: ―	―	2	Hepatectomy	3	41
			2nd: 6	Liver	5	Hepatectomy		
			3rd: 8	Liver	6	HAIC		
Suzuki, 2010^6)^	62, M	DP	1st: 7	Liver	3	Hepatectomy only	3	22
			2nd: 28	Liver	1			
			3rd: 39	Liver	1			
Minamiura, 2025^5)^	60, F	SPDP	1st: 27	Liver	1	Hepatectomy	2	22
			2nd: 36	Liver	1	mFFX, GEM →Hepatectomy		
Our case, 2026	22, M	DP	1st: 48	Local	―	GS	5	72
			2nd: 126	Liver	2	Hepatectomy + α		
			3rd: 137	Liver	1	Hepatectomy		
			4th: 142	Liver	Multiple	Hepatectomy		
			5th: 154	Liver	1	Hepatectomy		
			6th: 203	Liver	1	Hepatectomy		

α, resection of local recurrence and partial jejunectomy; DP, distal pancreatectomy; F, female; GEM, gemcitabine; GS, gemcitabine + s-1; HAIC, hepatic arterial infusion chemotherapy; M, male; mFFX, modified FOLFIRINOX; PACC, pancreatic acinar cell carcinoma; RFS, relapse free survival; SPDP, spleen-preserving distal pancreatectomy

In the present case, surgical resection was consistently chosen for liver metastases judged to be resectable, whereas GS therapy followed by S-1 therapy was introduced only for the unresectable local recurrence (year X + 4), which was deemed unresectable owing to its proximity to major mesenteric vessels. Surgery was prioritized for each subsequent liver recurrence because the lesions were solitary or limited in number, technically resectable with an adequate functional liver remnant, and amenable to R0 resection, which offers a more reliable chance of durable local control than chemotherapy alone for macroscopic disease. In addition, given the patient’s young age and the anticipated long clinical course, repeated surgery was considered preferable to long-term cumulative chemotherapy exposure. The favorable response of the unresectable local recurrence to GS/S-1 therapy suggests that the tumor retained a degree of chemosensitivity, and the fact that chemotherapy was not combined with resection for the resectable liver recurrences may be regarded as a limitation of the treatment approach in this case. Evidence regarding the role of perioperative chemotherapy in resectable recurrent PACC is limited. Although the present case demonstrates that long-term recurrence-free survival can be achieved with surgery alone, whether the addition of chemotherapy could have prolonged the recurrence-free intervals or reduced the risk of further recurrence remains uncertain. Further studies based on the accumulation of cases are warranted to clarify the role of perioperative chemotherapy in recurrent PACC.

In the present case, the patient was subsequently found to have FAP based on a germline *APC* pathogenic variant identified during long-term follow-up, prompting us to consider the biological relationship between FAP and PACC. FAP is an autosomal dominant disorder caused by germline pathogenic variants in the *APC* gene, which encodes a tumor suppressor protein that regulates the Wnt/β-catenin pathway.^8)^ It is also a systemic tumor syndrome associated with a wide range of malignancies beyond colorectal cancer, including gastric and duodenal cancer, desmoid tumors, and thyroid cancer. As noted above, a previous cohort study reported a cumulative incidence of PDAC of 0.7% by age 70 and a relative risk of 2.2 among patients with FAP,^2)^, suggesting an increased risk of PDAC in this population. In contrast, whether FAP also increases the risk of PACC remains unclear given the rarity of PACC. Notably, PACC itself frequently harbors alterations in the APC/β-catenin pathway, ^8,9)^ and reports of PACC arising in patients with FAP, including the present case, remain extremely rare, limited to those by Ohsato et al.^10)^ and Seket et al.^11)^ This molecular overlap raises the possibility of a biological association between FAP and PACC; however, the evidence remains preliminary and largely speculative, and further case accumulation and molecular investigation will be needed to clarify this relationship.

## CONCLUSIONS

We report a rare case of PACC arising in a patient with FAP, in which repeated hepatectomies for multiple recurrent liver metastases achieved long-term recurrence-free survival. Repeated hepatectomy for liver metastases from PACC may achieve long-term survival with appropriate case selection. In addition, this case raises the hypothesis that the APC/β-catenin pathway, shared between FAP and PACC, may be biologically involved in PACC tumorigenesis; however, this observation does not establish an epidemiological association between FAP and an increased risk of PACC, and further studies are needed to clarify this relationship.

## DECLARATIONS

### Use of artificial intelligence (AI) tools

During manuscript preparation, the corresponding author used Claude (Anthropic) and Gemini (Google) to assist with Japanese-to-English translation and improve the clarity of the English text. These tools were not used to generate, analyze, or interpret clinical data. The authors reviewed all AI-assisted content and take full responsibility for it.

### Availability of data and materials

The datasets supporting the conclusions of this article are included within the article.
